# Artificial intelligence for craniofacial and mandibular growth prediction: A systematic review

**DOI:** 10.1007/s00784-026-07190-2

**Published:** 2026-10-05

**Authors:** Alessandro Polizzi, Stefano Martina, Salvatore La Rosa, Rosalia Leonardi

**Affiliations:** 1 Department of General Surgery and Surgical-Medical Specialties, School of Dentistry, University of Catania, Via S.Sofia 78, 95124 Catania, Italy; 2 Department of Medicine, Surgery and Dentistry, University of Salerno, Via S. Allende, 84081 Baronissi, Italy

**Keywords:** Craniofacial growth, Orthodontics, Artificial intelligence, Prediction, Machine learning, Deep learning

## Abstract

**Objectives:**

The primary objective was to evaluate the predictive performance of artificial intelligence (AI) models for craniofacial and mandibular growth prediction. Secondary objectives were to assess methodological quality and identify research gaps.

**Materials and methods:**

Nine databases were searched through August 12, 2026, supplemented by manual searches. Human studies assessing AI-based models for craniofacial or mandibular growth prediction were included. Study characteristics, prediction intervals, AI approaches, performance metrics, and validation strategies were extracted. Risk of bias and applicability were assessed using PROBAST.

**Results:**

Twelve studies were included. Six assessed overall craniofacial growth, whereas six mandibular development. Prediction horizons ranged from ≤ 1.5 to approximately 10 years, with sample sizes of 33–639 participants. Predictive accuracy varied by anatomical region and methodological approach. Sex and malocclusion type were frequently used to define study populations but were not consistently incorporated as predictors, whereas ethnicity and skeletal maturation were rarely considered. Seven studies had overall high risk of bias and five unclear risk, mainly due to analytical limitations and inadequate validation.

**Conclusion:**

AI-based models show promising predictive performance; however, current evidence is insufficient to establish superiority over traditional statistical methods. Overall high or unclear risk of bias and substantial heterogeneity in study populations and datasets, prediction horizons, outcome definitions, and AI modelling approaches limit confidence in the findings. Standardized multicenter datasets and independent external validation are required before clinical implementation.

**Clinical relevance:**

AI-based growth prediction may support individualized orthodontic diagnosis and treatment timing, but current evidence does not support routine clinical use.

**Supplementary Information:**

The online version contains supplementary material available at https://doi.org/10.1007/s00784-026-07190-2.

## Introduction

Craniofacial growth is a significant topic in orthodontics, particularly in clinical practice, in that growth prediction assists orthodontists in formulating treatment plans and visualizing therapeutic outcomes to accomplish satisfying results for growing patients [1]. In fact, an accurate prediction of craniofacial growth in terms of magnitude and direction would offer substantial benefits in orthodontics, enhancing diagnosis and treatment planning [2].

Over the last century, craniofacial growth prediction has evolved from implant-based observations, mesh diagrams, forecast grids, templates, and visual treatment objectives [3–8] to statistical models designed to incorporate individual characteristics [1, 2, 9]. However, conventional approaches remain largely dependent on predefined growth assumptions or population averages and may therefore inadequately capture inter-individual variability [1, 2]. This limitation is particularly relevant because craniofacial growth reflects complex interactions among genetic, functional, and environmental factors [10, 11], making accurate patient-specific prediction a continuing challenge in orthodontics.

In the last few years, Artificial Intelligence (AI), Machine Learning (ML), and Deep Learning (DL) techniques have emerged as promising tools to address this challenge. These approaches utilize data-driven learning to identify complex patterns within high-dimensional datasets without requiring explicit growth equations [12]. The combined progress in processing capability, the availability of large datasets, and increasingly sophisticated ML techniques could allow AI to reach greater precision in identifying trends and forecasting outcomes. By recognizing nonlinear patterns and integrating large datasets, AI systems have the potential to predict patient-specific growth dynamics more accurately than conventional statistical approaches. Thus, in line with broader trends in modern healthcare, artificial intelligence may facilitate the transition from reliance on statistical averages to the generation of clinically individualized predictions. In this context, personalized orthodontics is expected to align with the wider medical paradigm of “P4 medicine,” which emphasizes the assumption that care is predictive, preventive, personalized, and participatory [13]. However, it remains unclear how consistently these models perform, which methodological limitations influence their reported performance, and whether the available evidence is sufficiently robust and generalizable for clinical application. Therefore, a systematic synthesis is needed to integrate evidence on predictive performance alongside assessments of methodological quality and applicability.

Accordingly, the primary objective of this systematic review was to evaluate the predictive performance of AI-based models for craniofacial and mandibular growth prediction. Secondary objectives were to assess the methodological quality and applicability of the available evidence and to identify the main research gaps that should be addressed before clinical implementation.

## Materials and methods

### Protocol and registration

This systematic review adhered to the Preferred Reporting Items for Systematic Reviews and Meta-Analyses (PRISMA) 2020 guidelines [14]. The protocol was registered in PROSPERO (CRD420251172592). During the conduct of the review, two methodological refinements were introduced relative to the registered protocol: the literature search was expanded to additional information sources to improve retrieval comprehensiveness, and PROBAST was used instead of the originally planned Newcastle–Ottawa Scale because it provides a framework specifically designed for studies developing or validating prediction models. These changes did not alter the review question, eligibility framework, outcomes of interest, or planned narrative synthesis, and the PROSPERO record has been updated accordingly.

### Selection process

A comprehensive search was conducted across nine electronic information sources: MEDLINE/PubMed, Cochrane CENTRAL, Web of Science Core Collection, Scopus, Virtual Health Library (VHL/BVS; non-MEDLINE sources), LIVIVO–ZB MED Search Portal for Life Sciences, IEEE Xplore, ProQuest Dissertations & Theses Global, and Cambridge Core. All searches were performed on August 12, 2026, without publication-date restrictions. Database-specific strategies and retrieval yields are reported in Table 1. Reference lists of eligible studies and Google Scholar were additionally screened to identify potentially relevant reports not retrieved through the electronic searches.

**Table 1:** Search Strategy for all the consulted databases

Scientific databases	Search strategy	Hits
MEDLINE—PubMed searched on August 12, 2026 www.ncbi.nlm.nih.gov/sites/entrez/	(craniofacial[Title/Abstract] OR facial[Title/Abstract] OR maxillofacial[Title/Abstract] OR mandib*[Title/Abstract] OR maxill*[Title/Abstract] OR jaw[Title/Abstract] OR cephalometr*[Title/Abstract] OR cephalogram*[Title/Abstract] OR orthodont*[Title/Abstract] OR malocclusion*[Title/Abstract]) AND (growth[Title/Abstract] OR development*[Title/Abstract] OR longitudinal[Title/Abstract] OR pubertal[Title/Abstract] OR postpubertal[Title/Abstract] OR "post-pubertal"[Title/Abstract]) AND (predict*[Title/Abstract] OR forecast*[Title/Abstract] OR prognos*[Title/Abstract]) AND ("Artificial Intelligence"[MeSH Terms] OR "Machine Learning"[MeSH Terms] OR "Neural Networks, Computer"[MeSH Terms] OR "artificial intelligence"[Title/Abstract] OR "machine learning"[Title/Abstract] OR "deep learning"[Title/Abstract] OR neural network*[Title/Abstract])	408
THE COCHRANE LIBRARY (Clinical Trial) searched on August 12, 2026 www.thecochranelibrary.com	((craniofacial OR facial OR maxillofacial OR mandib* OR maxill* OR jaw OR cephalometr* OR orthodont* OR malocclusion*) AND growth AND (predict* OR forecast* OR prognos*) AND ("artificial intelligence" OR "machine learning" OR "deep learning" OR "neural network" OR "neural networks" OR "random forest" OR "random forests" OR "support vector machine" OR "support vector machines" OR "artificial neural network" OR "artificial neural networks" OR "convolutional neural network" OR "convolutional neural networks")):ti,ab,kw	4
WEB OF SCIENCE CORE COLLECTION searched on August 12, 2026 https://www.webofscience.com/wos/woscc/smart-search	TS = ((craniofacial OR facial OR maxillofacial OR mandib* OR maxill* OR jaw OR cephalometr* OR orthodont* OR malocclusion*) AND growth AND (predict* OR forecast* OR prognos*) AND ("artificial intelligence" OR "machine learning" OR "deep learning" OR "neural network*" OR "random forest*" OR "support vector machine*" OR “artificial neural network” OR “convolutional neural network”))	145
SCOPUS searched on August 12, 2026 www.scopus.com	TITLE-ABS((craniofacial OR facial OR maxillofacial OR mandib* OR maxill*) AND growth AND predict* AND ("artificial intelligence" OR "machine learning" OR "deep learning" OR "neural network*"))	135
Virtual Health Library (VHL/BVS Regional Portal) – non-MEDLINE sources searched on August 12, 2026 http://bvsalud.org/en/	tw:((craniofacial OR facial OR maxillofacial OR mandib* OR maxill* OR jaw OR cephalometr* OR orthodont* OR malocclusion*) AND growth AND (predict* OR forecast* OR prognos*) AND ("artificial intelligence" OR "machine learning" OR "deep learning" OR "neural network" OR "neural networks" OR "random forest" OR "random forests" OR "support vector machine" OR "support vector machines" OR "artificial neural network" OR "artificial neural networks" OR "convolutional neural network" OR "convolutional neural networks"))	27
LIVIVO – ZB MED Search Portal for Life Sciences (BASE and non-MEDLINE sources) searched on August 12, 2026 https://www.livivo.de/app/search/search	(craniofacial OR facial OR maxillofacial OR mandibular OR maxillary OR jaw OR cephalometric OR orthodontic OR malocclusion) AND growth AND (predict OR prediction OR forecast OR prognosis) AND ("artificial intelligence" OR "machine learning" OR "deep learning" OR "neural network" OR "random forest" OR "support vector machine" OR "artificial neural network" OR "convolutional neural network")	265
IEEE XPLORE searched on August 12, 2026 https://ieeexplore.ieee.org/Xplore/home.jsp	(craniofacial OR facial OR maxillofacial OR mandib* OR maxill* OR jaw OR cephalometr* OR orthodont* OR malocclusion*) AND growth AND (predict* OR forecast* OR prognos*) AND ("artificial intelligence" OR "machine learning" OR "deep learning" OR "neural network" OR "neural networks" OR "random forest" OR "random forests" OR "support vector machine" OR "support vector machines" OR "artificial neural network" OR "artificial neural networks" OR "convolutional neural network" OR "convolutional neural networks")	117
PROQUEST DISSERTATIONS & THESES (UMI / PQDT GLOBAL) searched on August 12, 2026 https://www.proquest.com/	(craniofacial OR facial OR maxillofacial OR mandibular OR maxillary OR cephalometric OR orthodontic OR malocclusion) AND ("growth prediction" OR "predicting growth" OR "growth forecasting" OR "growth forecast") AND ("artificial intelligence" OR "machine learning" OR "deep learning" OR "neural network" OR "neural networks" OR "random forest" OR "random forests" OR "support vector machine" OR "support vector machines" OR "artificial neural network" OR "artificial neural networks" OR "convolutional neural network" OR "convolutional neural networks")	7
CAMBRIDGE CORE searched on August 12, 2026 https://www.cambridge.org/core/	(jaw OR maxilla OR mandible OR craniofacial OR facial OR mandibular OR maxillary) AND (growth) AND (artificial intelligence OR machine learning OR deep learning OR neural network* OR artificial neural network* OR convolutional neural network*)	43
TOTAL		**1151**

### Eligibility criteria

Studies were eligible if they included human participants with longitudinal observations of natural or untreated craniofacial or mandibular growth and applied AI, ML, or DL algorithms to predict subsequent growth over time. For study classification, an AI-based approach was defined as one including at least one data-driven machine-learning or deep-learning model trained on empirical input data to learn a predictive relationship with subsequent craniofacial or mandibular growth outcomes. Computerized methods based exclusively on predefined growth equations, deterministic rules, conventional statistical models without a machine-learning component, or software implementations of established prediction methods were not considered AI-based. No a priori restrictions were imposed on chronological age, sex, ethnicity, skeletal class, malocclusion type, or skeletal maturation stage. Studies were required to report at least one quantitative measure of predictive performance. Eligibility was restricted to studies available in English.

Investigations employing AI solely for diagnosis or classification without longitudinal prediction of craniofacial growth were excluded. Studies whose predictive target was treatment response, treatment choice, or another outcome rather than natural craniofacial growth were also excluded. Case reports, literature reviews, editorials, conference abstracts lacking full data, and studies not presenting quantitative predictive results were excluded.

### Data extraction and synthesis

Exportable records were imported into EndNote (Clarivate Analytics, 2025), and duplicates were removed. Two reviewers independently screened titles and abstracts for potential eligibility. Full-text articles were then assessed against the inclusion criteria. Disagreements were resolved by discussion, and a third reviewer was consulted when consensus could not be reached.

The following variables were collected: author, year of publication, aim, data source, sample size, predictor variables, growth examination interval, predicted time output, AI methods, model validation strategy, results and conclusion. Given the substantial clinical and methodological heterogeneity across the included studies, a quantitative meta-analysis was not considered appropriate and findings were synthesized narratively. Study characteristics and results were organized in structured tables and compared according to population and data source, anatomical prediction target, growth examination and prediction interval, AI modelling approach, validation strategy, and reported performance metrics. Because outcome definitions and performance measures varied considerably among studies, results were retained in their originally reported metrics and were not statistically pooled or transformed into a common effect measure. The synthesis focused on identifying patterns in predictive performance, methodological differences, validation approaches, and recurrent limitations across studies.

### Risk of bias assessment

The risk of bias and applicability of the included studies were assessed using the Prediction model Risk Of Bias ASsessment Tool (PROBAST) [15], which evaluates four domains: Participants, Predictors, Outcome, and Analysis. Two reviewers independently assessed each study according to the PROBAST signaling questions, assigning a low, high, or unclear risk of bias. Discrepancies were resolved through discussion, and when necessary, consultation with a third reviewer. An overall risk of bias judgment, was assigned to each study following PROBAST guidance, whereby a study was considered at high risk of bias if at least one domain was judged to be at high risk. The applicability of each study to the review question was also evaluated across the three relevant domains (Participants, Predictors, and Outcome), considering whether the study population, predictors, and outcomes were representative of clinically relevant craniofacial growth prediction scenarios. No ad hoc modifications were made to the original PROBAST signaling questions or domain-level judgment criteria. For AI- and ML-based models, particular attention was given within the relevant signaling questions to sample size relative to model complexity, overfitting and optimism, training/validation/testing procedures, cross-validation strategies, and independence of test data.

## Results

**Fig. 1:**
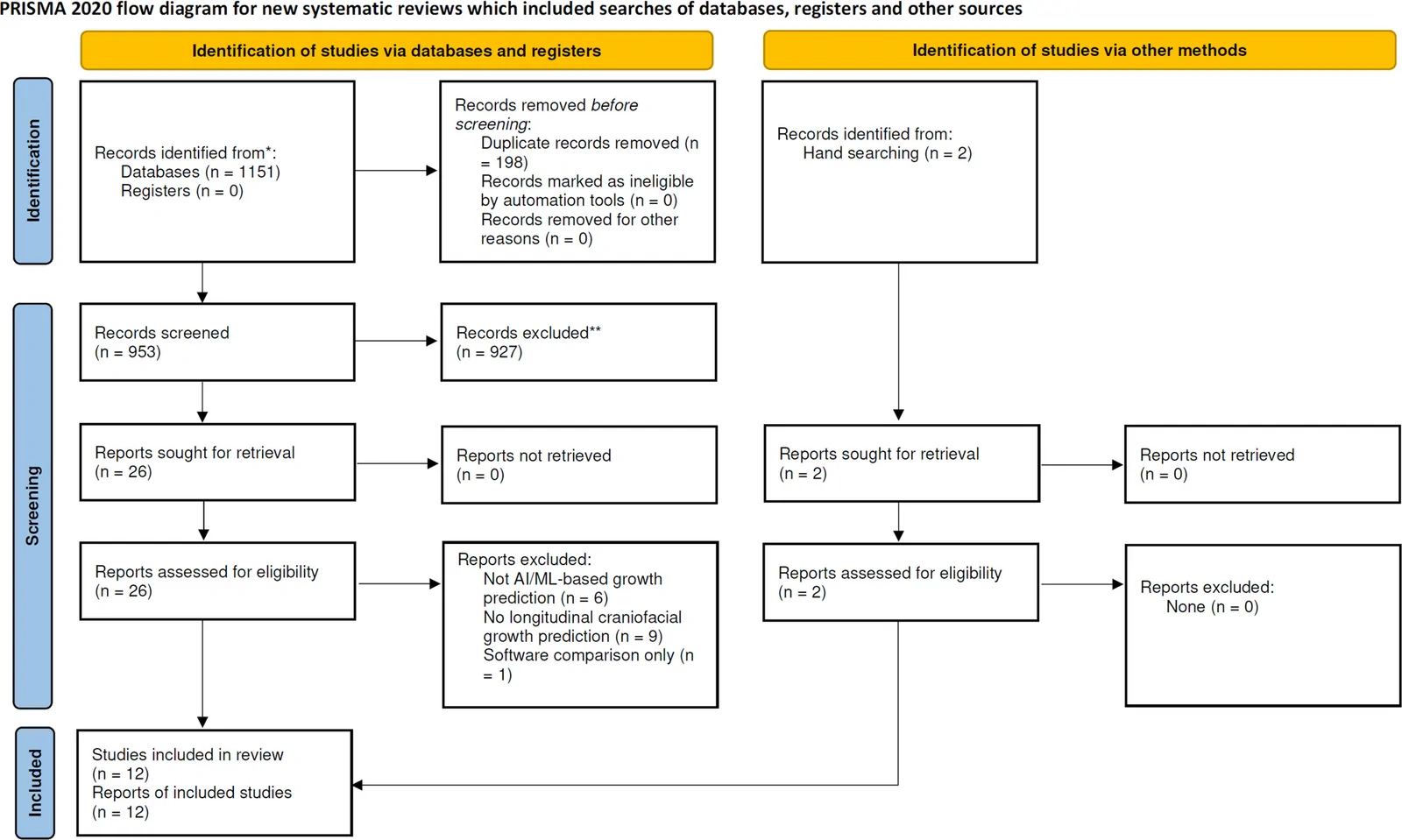
PRISMA 2020 flow chart of the study-selection process

The electronic searches yielded 1,151 records. After removal of 198 duplicates, 953 records were screened by title and abstract, of which 927 were excluded. Twenty-six reports identified through electronic searching were assessed in full text; 16 were excluded because they did not use an eligible AI/ML-based growth-prediction approach (*n* = 6), did not evaluate longitudinal craniofacial or mandibular growth prediction (*n* = 9), or represented a software-comparison study without an eligible predictive model (*n* = 1). Ten studies from the electronic search met the eligibility criteria. Manual searching identified two additional reports, both of which were assessed and included, resulting in 12 studies in the qualitative synthesis. The study-selection process is summarized in Fig. 1. Individual reasons for full-text exclusion are reported in Supplementary Table 1.

### Qualitative synthesis of the studies

The characteristics and main findings of the included studies are presented in Table 2. All the published papers aimed to forecast either the whole craniofacial growth or just mandibular growth with the aid of AI. The sample sizes varied widely, ranging from 33 to 639 participants, with approximately half of the studies using the longitudinal datasets from the American Association of Orthodontists Foundation (AAOF) Legacy Collection [16], while the remaining studies relied on independently collected longitudinal samples.

**Table 2:** Data extracted from studies. AAOF: American Association of Orthodontists Foundation; CNN: Convolutional Neural Network; CV: cross-validation; DNN: deep neural network; GNN: Graph Neural Network; Lasso: Least Absolute Shrinkage and Selection Operator; LOOCV: leave-one-out cross-validation; MAE: mean absolute error; ME: mean error; ML: machine learning; RMSE: root mean square error

Author Year	AIM	Dataset	Sample Size	Predictor variables	Growth examination interval	Predicted Time output (Age)	AI method	Validation strategy	Results	Conclusions
Jiwa 2020 [17]	Mandibular growth prediction	Forsyth Moorrees twin study	101 (81 training 20 test)	52 males 49 females Caucasian descent Class I malocclusion Class II malocclusion Class III malocclusion	T1 = 7 yrs T2-T3 = 8–17 yrs	2-years	-Multilayer Perceptron	Hold-out test set	Landmark mean Error range 2,47 mm-6,09 mm	Multilayer Perceptron is not a on par with Ricketts’ growth prediction for a 2 years forecast period
Kaźmierczak 2021 [27]	Mandibular growth prediction	AAOF Legacy Collection Niddetal growth study	639 (608 training 31 test)	N.R	T1 = 9.06 yrs T2 = 12.07 yrs T3 = 17.41 yrs	N.R	-Neural Networks -Random Forest -Logistic regression -XGBoost -Support Vector Machine	Repeated fivefold CV	Best Accuracy range 71% -75%	Not every model tested obtained the same accuracy. The accuracy varied between 71 and 75% The best models achieved better results depending on the predicted variable
Kim E., 2023 [23]	Craniofacial growth prediction	Dental University of Kanagawa Japan	59 (no fixed train/test split)	27 males 32 females	T1 = 6 yrs T2 = 12 yrs T3 = 13 yrs	7 year	-Lasso -Radial Basis Function Network -Multilayer Perceptron -Gradient-Boosted Decision Tree -Support Vector Regression	tenfold CV	Root Men Square Error (RMSE) Landmarks 0,67 mm-12,77 mm Cephalometric measuments 0,74 mm – 7,57 mm 0,74^—15,54^	Lasso had the smallest error for all values and parameters The highest prediction accuracies when predicting skeletal linear and angular parameters for 13-year-olds were 97.87% and 94.45%, respectively
Parrish M., 2023 [19]	Mandibular growth prediction	AAOF Legacy Collection	176 (140 training 36 test)	176 Females Angle Class I	T1 = 10,05 yrs T2 = 11,98 yrs T3 = 13,85 yrs	2 years 4 years	-XGBoost, -Random Forest -Lasso -Ridge -Linear Regression -Support Vector Regression -Multilayer Perceptron	Validation + hold-out test set	2 years MAE range 2,78 to 5,40 mm 0,88^-1,48^ 4 Years MAE range 3,21 to 4,00 mm 0,19^-1,66^	Tested ML models were able to predict post-pubertal mandibular length within 3 mm and Y-axis within 1 degree
Wood 2023[20]	Mandibular growth prediction	AAOF Legacy Collection	163 (114 training 49 test)	163 males Angle Class I	T1 = 11.85 yrs T2 = 13.82 yrs T3 = 15.80 yrs	4 years prediction	-Least Squares -Ridge -Lasso -Elastic Net, -XGBoost -Random Forest -Neural Network	Hold-out test set	MAE range 2 years 3.31 mm—5.47 mm 1.12^—2.29^ 4 years 2,32 mm -5,28 mm 1,25–1,72^	No significant difference was found among the accuracies of the techniques, except the least square method had a significantly larger error than all others in predicting the Y axis of growth
Zakhar 2023 [21]	Mandibular growth prediction	AAOF Legacy Collection	123 (92 training 31 test)	123 boys Class II malocclusion	T1 = 12yrs T2 = 14.1 yrs T3 = 15.9 yrs	2 years prediction 4 years prediction	-XGBoost, -Random Forest -Lasso, -Ridge Regression -Linear regression -Support Vector Regression -Multilayer Perceptron	Hold-out test set	MAE range 2 years 2.11 mm—6.07 mm 0.85^—2.74^ 4 years 2.32 mm—5.28 mm 1,25^—1,72^	The tested ML algorithms successfully predicted the post-pubertal mandibular length within a range of 2.5 mm and the y-axis within 1
Zhang J.N., 2023 [22]	Mandibular growth prediction	Sir Run Run Shaw Hospital, Zhejiang University School of Medicine China	296 256 training [40 testing (20 prognatic mandible, 20 normal mandible)]	142 males 154 females - Anterior crossbite; - Class III Class I molar relationship; - Airway morphology	T1 = 8,08 yrs T2 = 13,92 yrs	6 years prediction	-Deep Convolutional Neural Network	tenfold CV + hold-out test set	CNN prediction accuracy 85%, compared to 54.2% junior orthodontists	The deep-learning CNN model could predict the growth trend of the mandible in anterior crossbite children with relatively high accuracy using cephalometric images
Larkin 2024 [18]	Craniofacial growth prediction	Okajama University Japan	198 (161 training 17 validation 20 test)	Gender N.R Class I malocclusion	T1 = 8 yrs T2 = 10 yrs	2 years prediction	-Convolutional Neural Network	Train/validation/test split	ME hard tissue 0,03–1,99 mm M.E. soft tissue 1,01–2,19	Short-term growth prediction might be possible in preadolescent patients with Class I malocclusion using the CNN algorithm for a 2-year growth interval
Moon 2024 [9]	Craniofacial growth prediction	Seoul National University Dental Hospital Seoul Korea	410 (no fixed train/test split)	174 males 236 females	T1 = 11.5 yrs T2 = 15.3 yr	4 years prediction	-TabNet Deep Neural Network	LOOCV	Error Range skeletal landmark 1.7 mm—3.7 mm soft-tissue landmark 2.1 mm—4.3 mm	The TabNet AI algorithm predicted growth more accurately than the multivariate partial least squares regression method
Myers M., 2025 [24]	Craniofacial growth prediction	AAOF Legacy Collection	301 (240 training 61 test)	Class I malocclusion Class II Class III	T1 = 11 yrs T2 = 18 yrs	8 yrs	Lasso, Random Forest, Support Vector Regression	fivefold CV + test set	MAE range 1,26 mm-4,09 mm 1,36^-5,39^	ML models demonstrated the ability to predict post-pubertal values. with a clinically acceptable margin of 2 mm or 2°
Roseth 2025 [25]	Craniofacial growth prediction	Mathews collection from AAOF Legacy Collection	33 (no fixed train/test split)	12 males 21 females Class I Class II	T1 = 7.37 yrs T2 = 15.86 yrs	8,49 yrs	TabNet Deep Neural Network (DNN)	LOOCV	Euclidean distance error range: skeletal landmark 1.21 mm—1.99 mm soft-tissue landmark 1.14 mm—3.45 mm	AI proved to be a valuable growth prediction method, with clinically acceptable prediction errors averaging 1.49 mm for hard tissue landmarks and 1.71 mm for soft tissue landmarks
Statie 2026 [26]	Craniofacial growth prediction	Florence/Michigan longitudinal untreated Class III cohort	144 (116 train; 28 test) short-term (ST) test: 16; long-term (LT) test: 13	65 M; 79 F; Caucasian; untreated Class III 16 landmark X/Y coordinates	T0: 10.0 ± 3.7 yrs Last: 13.8 ± 2.7 yrs	ST: ≤ 1.5 y (1.1 ± 0.3) LT: > 3 y (5.1 ± 2.0; 3.2–10.3)	Graph Neural Network (GNN)	fivefold CV + hold-out test set	Euclidean distance error range: skeletal landmarks ST: 0.7 mm–2.6 mm LT: 1.0 mm–4.5 mm	GNN was accurate for most landmarks, with the largest errors at mandibular landmarks, especially in the long-term set

Nine studies used a fixed training/test or training/validation/test allocation, whereas three used cross-validation without a fixed split. Predictor variables, such as ethnicity, sex, malocclusion type, facial growth pattern, skeletal maturation, and oral habits, were not investigated or were poorly characterized in more than half of the studies.

Forecast intervals differed across studies, spanning from a short-term horizon [17, 18] of approximately two years, medium-term four years [1, 19–21] to long-term projections extending up to 10 years [22–26].

A wide range of AI methodologies were implemented. Traditional ML algorithms such as Lasso, Ridge regression, Random Forest, XGBoost, multilayer Perceptron, linear regression and Support Vector Regression (SVR) were applied in several studies [17, 19–22, 24, 27], while deep learning architectures included Convolutional Neural Networks (CNNs), Graph Neural Network (GNN), and TabNet-based models for feature extraction and longitudinal prediction [9, 18, 22, 25, 26].

Predictive performance metrics such as Mean Absolute Error, Mean Error, Root Mean Square Error, Euclidean Distance differences, and Percentage of prediction accuracy were consistently reported across studies. However, the performance metrics reported across the studies were too heterogeneous to permit an easy, meaningful comparison of the results.

### Risk of bias results

According to the PROBAST assessment (Table 3), 7 of the 12 included studies were judged at overall high risk of bias [17, 21, 23–27], and 5 were rated as unclear risk [9, 18–20, 22].

**Table 3:**
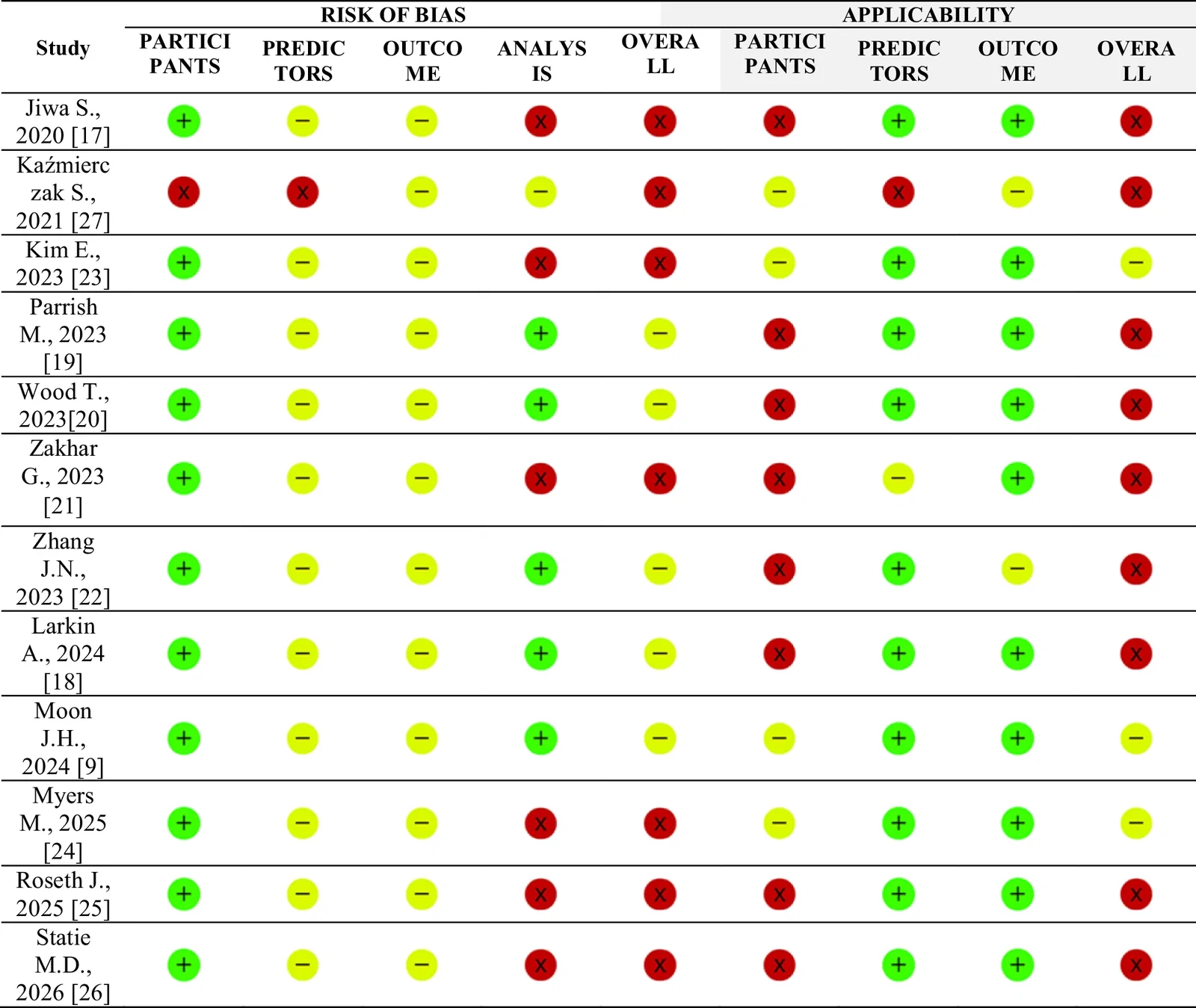
PROBAST risk of bias and applicability concerns assessment. High risk, Unclear risk, Low risk

Risk of bias in the participants' domain was generally low. Most studies used established longitudinal growth archives or well-defined retrospective cohorts with appropriate inclusion and exclusion criteria. However, one study intentionally expanded age inclusion criteria to increase sample size, introducing heterogeneity in growth stage among patients [27]. Applicability concerns were more prominent than internal bias, largely due to highly selected or demographically restricted samples [17, 21, 22, 25, 26].

Risk of bias in the Predictors domain was rated as unclear in 11 studies [9, 17–26] and high in one study [27], primarily because blinding of predictor assessment to subsequent outcome information was not reported. The Outcome domain was rated as unclear in all included studies largely because it was not reported whether outcome assessment at the later timepoint was performed without knowledge of baseline predictor information. Nevertheless, outcome definitions were generally appropriate and prespecified, including continuous cephalometric measures [19, 20], landmark coordinate prediction [18, 25], or clinically defined growth classifications [22].

Although risk of bias in the Participants domain was generally low (reflecting appropriate participant selection and data sources within the individual studies), applicability concerns were concentrated primarily in this domain because several studies relied on highly selected populations defined by sex, ethnicity, skeletal class, or historical growth-archive membership. In contrast, applicability concerns for Predictors and Outcome were generally low because the variables and future growth targets were clinically relevant to orthodontic growth prediction.

The analysis domain was the most critical source of bias. High risk was identified when sample size was small relative to model dimensionality [17, 23, 25, 26], when internal validation procedures were insufficient to control optimism [21], or when cross-validation strategies were potentially misapplied [24]. Several studies appropriately used internal cross-validation and independent test sets [9, 18, 19], which mitigated concerns regarding overfitting.

Individual signaling-question judgments are reported in Supplementary Table 2.

## Discussion

Accurate prediction of craniofacial growth remains a fundamental pillar in orthodontics, as treatment timing and modality are highly dependent on individual growth potential. Despite decades of research, growth prediction remains challenging due to the complex interplay of genetic, environmental, and functional factors that shape the craniofacial complex.

In fact, there are several drawbacks on the facial growth prediction methods proposed till now, in that, whether the oldest prediction methods are based on predefined assumptions regarding growth direction and magnitude, the statistical methods for predicting craniofacial growth are limited by the high inter-individual variability, which may not reflect a specific patient.

Recent advances in AI, particularly ML and DL techniques, have introduced new opportunities for the development of data-driven growth predictive models, capable of integrating large and heterogeneous datasets. Accordingly, AI-based approaches to craniofacial growth prediction have recently been increasingly explored. However, a comprehensive critical evaluation of the current level of evidence and of its applicability to orthodontic practice has not yet been carried out, and the overall methodological quality and clinical applicability of these models remain unclear. Therefore, the aim of the present review was to critically appraise the current literature on AI-driven craniofacial growth prediction and to assess its potential relevance for orthodontics.

The analysis of the literature suggests that AI holds promising potential for predicting craniofacial growth. Predictive accuracy varied by anatomical region and methodological approach used. Some individual studies reported differences in prediction errors across anatomical regions and measurements [9, 23, 25, 26]; however, substantial differences in study populations, prediction horizons, outcomes, and model architectures preclude conclusions regarding the superiority of any specific AI approach. Soft-tissue and mandibular landmark predictions frequently showed greater variability or larger errors in individual studies [17, 24–26]. However, the substantial heterogeneity in datasets and study designs makes direct comparison across investigations difficult and precludes definitive conclusions.

Many concerns deal with the number of subjects included in the longitudinal serial sample, ethnicities, predicted time / growth examination interval, outcome definitions, and performance metrics, which prevent a direct comparison among investigations and a generalizability of the proposed models.

As far as the dataset is concerned, it should be underlined that several investigations relied on small samples [23, 25, 26].

At this regard, for the sake of clarity, it has been suggested that the performance of AI could improve as the size of the training data increases [28]. However, according to other studies [29, 30], it could introduce individual variability, thereby reducing accuracy.

To train and test the AI algorithms, approximately half of the studies used the AAOF database [16, 19–21, 24, 25, 27], which is composed primarily of patients of European ancestry, while other studies relied on Asian populations [9, 18, 22, 23]. These population differences limited direct cross-study comparison and, in the absence of external validation across populations, precluded conclusions regarding the generalizability of the models across different ethnic groups. Therefore, predictive performance observed in ethnically restricted, sex-specific, or historical archive-based samples cannot be assumed to generalize to broader orthodontic populations. This lack of population representativeness and external validation represents a major barrier to clinical implementation. Moreover, in some studies, the number of subjects used for algorithm training versus testing was not specified. Only a few studies employed a broadly representative population sample [9, 24], whereas in others the inclusion criteria were highly restrictive, for example, by developing the algorithm exclusively on one sex [19–21]. Importantly, the generally low risk of bias observed in the PROBAST Participants domain should not be interpreted as evidence of broad generalizability, as several studies nevertheless showed substantial applicability concerns due to restricted or highly selected study populations. Growth examination interval and predicted time output also varied considerably: some studies included samples with an age range of eight years [24, 25], others four years [9, 20], and still others only two years [18]. In the latter case, it was not even specified whether the patients were assessed during their supposed peak growth period.

The few studies directly comparing AI-based models with established statistical methods demonstrated a superior performance of ML over Partial Least Squares (PLS) regression. [9, 25]; however, these findings are insufficient to establish general superiority.

Some studies evaluated comprehensive craniofacial growth patterns [9, 18, 23–26], whereas others focused exclusively on mandibular development, without assessing maxillary changes [17, 19–22, 27]. Only a minority clearly stratified patients according to skeletal Class I, II, or III [17, 24], despite well-documented differences in growth trajectories among these groups [31]. The absence of skeletal pattern stratification may partly explain variability in reported accuracy. Accordingly, without external validation across different skeletal patterns, the performance of models developed in restricted Class I, II, or III populations cannot be extrapolated to patients with different craniofacial growth characteristics.

Taken together, these findings highlight that AI advancements in craniofacial growth prediction are limited by substantial heterogeneity of the included studies in sample selection, anatomical focus, prediction interval, and comparator use. Few studies reported sufficiently detailed training–testing strategies to fully assess model robustness. Most critically, independent external validation was absent across all included studies, preventing assessment of model transportability to populations differing in ethnicity, sex, skeletal pattern, or clinical setting. These methodological limitations are reflected in the PROBAST assessment, with seven studies judged at overall high risk of bias and the remaining five at unclear risk, thereby limiting confidence in the reported predictive performance and raising the possibility of overly optimistic estimates. Small samples relative to model complexity and limitations in validation strategies may have increased the risk of overfitting and optimistic performance estimates, while highly selected populations further restrict generalizability. More importantly, many studies relied on retrospective growth archives lacking detailed information on skeletal maturation stage, ethnicity, familial growth patterns, or environmental determinants, since prospective longitudinal growth datasets are ethically difficult to obtain. From a broader biological perspective, craniofacial growth is influenced by genetic, local, and environmental determinants, with different components of the craniofacial complex showing varying susceptibility to these influences [32, 33]. Although these factors were not systematically investigated as determinants of model performance in the included studies, their incomplete characterization may represent an additional source of unexplained variability. Accordingly, the available evidence should be regarded as preliminary and interpreted cautiously before conclusions regarding generalizable clinical performance can be drawn.

For these reasons, future research should prioritize harmonized multicenter datasets incorporating skeletal maturation indicators (e.g., cervical vertebral maturation [34], if hand-wrist X-ray is not available), broader demographic representation, and transparent reporting of model development and validation procedures. Among these priorities, independent external validation and the development of standardized multicenter datasets should be considered the most critical methodological requirements. Moreover, beyond methodological challenges, ethical considerations remain central to the integration of AI into orthodontic practice, including issues of data governance, algorithmic transparency, informed consent, and accountability for clinical decisions supported by black-box models [35].

Accordingly, the P4 framework remains an aspirational direction, whose clinical translation will depend on the development and external validation of more robust, generalizable, and clinically meaningful AI-based growth prediction models, with algorithmic outputs supporting, rather than replacing, biologically informed clinical decision-making.

### Limitations of this systematic review

This systematic review has several limitations. Substantial clinical and methodological heterogeneity across the included studies, particularly in study populations, prediction targets, prediction horizons, AI modelling approaches, validation strategies, and performance metrics, precluded quantitative meta-analysis and limited direct cross-study comparisons. Eligibility was restricted to studies available in English, which may have introduced language bias. Moreover, the evidence base comprised only 12 studies, all of which were judged at overall high or unclear risk of bias, thereby limiting confidence in the conclusions that can be drawn from the available literature. Accordingly, the synthesis necessarily remained qualitative, and comparative findings across models should be interpreted cautiously.

## Conclusions

- Available studies suggest that AI-based models can achieve promising predictive performance for craniofacial and mandibular growth prediction; however, the limited number of direct comparisons with conventional methods does not permit firm conclusions regarding their superiority.
- Confidence in these findings remains limited because all the studies were judged at overall high or unclear risk of bias, together with substantial heterogeneity in study populations, datasets, prediction horizons, and modelling approaches.
- Before clinical implementation can be considered, future studies with independent external validation and the development of standardized multicenter datasets, supported by transparent model development and validation procedures and more representative study populations are needed.

## Supplementary Information

Below is the link to the electronic supplementary material.

Supplementary file1 (DOCX 20 KB)

Supplementary file2 (DOCX 34 KB)

Supplementary file3 (DOCX 270 KB)

## Data Availability

No datasets were generated or analysed during the current study.
