# Supplementary material for: Artificial intelligence for craniofacial and mandibular growth prediction: A systematic review
Source: Clin Oral Investig. 2026 Oct 5;30(10):507. doi: 10.1007/s00784-026-07190-2 (PMC13635025; doi:10.1007/s00784-026-07190-2)
Supplement: Supplementary file 1 [file 784_2026_7190_MOESM1_ESM.docx]

| **Article** | **Reason for exclusion** |
| --- | --- |
| **Sherwood R.J., et al**. Bayesian approach to longitudinal craniofacial growth: the craniofacial growth consortium study.  The Anatomical Record 2021; 304 (5): 991-1019. | Not AI/ML-based growth prediction |
| **Auconi P. et al.** Forecasting craniofacial growth in individuals with class III malocclusion by computational modelling.  European Journal of Orthodontics 2014; 36 (2): 207-216. | Not AI/ML-based growth prediction |
| **Moon J-H et al.** Evaluation of an individualized facial growth prediction model based on the multivariate partial least squares method. Angle Orthodontist 2022; 92 (6): 705-713. | Not AI/ML-based growth prediction |
| **Chvatal B.A. et al.** Development and testing of multilevel models for longitudinal craniofacial growth prediction. American Journal of Orthodontics and Dentofacial Orthopedics 2005; 128 (1): 45-56. | Not AI/ML-based growth prediction |
| **Volovic J. et al.** A novel machine learning model for predicting orthodontic treatment duration. Diagnostics 2023; 13 (17): 2740. | No longitudinal craniofacial or mandibular growth prediction |
| **Niño-Sandoval T.C. et al.** Use of automated learning techniques for predicting mandibular morphology in skeletal class I, II and III. Forensic Science International 2017; 281: 187-e1. | No longitudinal craniofacial or mandibular growth prediction |
| **Prasad J. et al.** Machine learning predictive model as clinical decision support system in orthodontic treatment planning. Dentistry Journal 2022; 11 (1) 1. | No longitudinal craniofacial or mandibular growth prediction |
| **Park J.H. et al.** Use of artificial intelligence to predict outcomes of nonextraction treatment of Class II malocclusions. Seminars in Orthodontics 2021; 27 (2): 87-95 | No longitudinal craniofacial or mandibular growth prediction |
| **Hersberger-Zurfluh M.A. et al.** Genetic and environmental components of vertical growth in mono- and dizygotic twins up to 15-18 years of age. Angle Orthodontist 2021; 91(3): 384-390. | Not AI/ML-based growth prediction |
| **Yamada S. et al.** Accuracy of Artificial Intelligence in Predicting the Treatment Effects of Headgear and/or Functional Appliance on the Maxillo-Mandibular Growth in Preadolescent Patients With Skeletal Class II Malocclusion. Orthodontics and Craniofacial Research. 2025; 28 (6): 955-961. | No longitudinal craniofacial or mandibular growth prediction |
| **Kumar M. et al.** Predicting treatment pathways in Class II malocclusion patients using machine learning: A comparative study of four algorithms for classifying camouflage, growth modulation, and surgical decisions. International Orthodontics 2025; 24 (1): 101070. | No longitudinal craniofacial or mandibular growth prediction |
| **Sagun M. et al.** Evaluation of Ricketts' and Bolton's growth prediction algorithms embedded in two diagnostic imaging and cephalometric software. Journal of the World Federation of Orthodontics 2015; 4 (4): 146-150 | Software comparison only |
| **Hu Z. et al.** Quantification of Craniofacial Growth Pattern Based on Deep Learning. Bioengineering 2026; 13 (3): 277. | No longitudinal craniofacial or mandibular growth prediction |
| **Oh H. et al.** Craniofacial growth percentile curves: A clinical tool from the Craniofacial Growth Consortium Study. American Journal of Orthodontics and Dentofacial Orthopedics 2026; 169 (6): 720-734. | Not AI/ML-based growth prediction |
| **Ashkan A. et al.** Feasibility of predicting vertical cephalometric angles from panoramic radiographs using deep learning. International Orthodontics 2026; 24 (3): 101156. | No longitudinal craniofacial or mandibular growth prediction |
| **Guo M. et al.** Prediction of skeletal maturity using machine learning based on multiple biological indicators. American Journal of Orthodontics and Dentofacial Orthopedics 2026; 169 (5): 629-639.e1. | No longitudinal craniofacial or mandibular growth prediction |

**Supplementary Table 1**. Articles excluded after full-text evaluation
