# Supplementary material for: Artificial intelligence for craniofacial and mandibular growth prediction: A systematic review
Source: Clin Oral Investig. 2026 Oct 5;30(10):507. doi: 10.1007/s00784-026-07190-2 (PMC13635025; doi:10.1007/s00784-026-07190-2)
Supplement: Supplementary file 2 [file 784_2026_7190_MOESM2_ESM.docx]

**Supplementary Table 2. PROBAST signaling-question, domain-level risk-of-bias, and applicability judgments.**

| **PROBAST item** | **Jiwa 2020 [17]** | **Kaźmierczak 2021 [27]** | **Kim 2023 [23]** | **Parrish 2023 [19]** | **Wood 2023 [20]** | **Zakhar 2023 [21]** | **Zhang 2023 [22]** | **Larkin 2024 [18]** | **Moon 2024 [9]** | **Myers 2025 [24]** | **Roseth 2025 [25]** | **Statie 2026 [26]** |
| --- | --- | --- | --- | --- | --- | --- | --- | --- | --- | --- | --- | --- |
| **Domain 1 - Participants** | | | | | | | | | | | | |
| 1.1 Were appropriate data sources used? | Y | PY | PY | Y | Y | Y | Y | Y | Y | Y | Y | Y |
| 1.2 Were all inclusions and exclusions appropriate? | PY | PN | Y | Y | Y | PY | PY | PY | PY | Y | PY | PY |
| **Domain 1 risk of bias** | **Low** | **High** | **Low** | **Low** | **Low** | **Low** | **Low** | **Low** | **Low** | **Low** | **Low** | **Low** |
| **Applicability - Participants** | **High** | **Unclear** | **Unclear** | **High** | **High** | **High** | **High** | **High** | **Unclear** | **Unclear** | **High** | **High** |
| **Domain 2 - Predictors** | | | | | | | | | | | | |
| 2.1 Were predictors defined and assessed in a similar way for all participants? | Y | PY | PY | Y | Y | Y | Y | Y | Y | Y | Y | Y |
| 2.2 Were predictor assessments made without knowledge of outcome data? | NI | NI | NI | NI | NI | NI | NI | NI | NI | NI | NI | NI |
| 2.3 Are all predictors available at the time the model is intended to be used? | Y | PN | PY | PY | PY | NI | Y | Y | Y | Y | Y | Y |
| **Domain 2 risk of bias** | **Unclear** | **High** | **Unclear** | **Unclear** | **Unclear** | **Unclear** | **Unclear** | **Unclear** | **Unclear** | **Unclear** | **Unclear** | **Unclear** |
| **Applicability - Predictors** | **Low** | **High** | **Low** | **Low** | **Low** | **Unclear** | **Low** | **Low** | **Low** | **Low** | **Low** | **Low** |
| **Domain 3 - Outcome** | | | | | | | | | | | | |
| 3.1 Was the outcome defined appropriately? | Y | PY | Y | Y | Y | Y | Y | Y | Y | Y | Y | Y |
| 3.2 Was a prespecified or standard outcome definition used? | Y | PY | PY | Y | Y | Y | PY | Y | Y | Y | Y | Y |
| 3.3 Were predictors excluded from the outcome definition? | Y | Y | Y | Y | Y | Y | Y | Y | Y | Y | Y | Y |
| 3.4 Was the outcome defined and determined in a similar way for all participants? | Y | Y | PY | Y | Y | Y | Y | Y | Y | Y | Y | Y |
| 3.5 Was the outcome determined without knowledge of predictor information? | NI | NI | NI | NI | NI | NI | NI | NI | NI | NI | NI | NI |
| 3.6 Was the time interval between predictor assessment and outcome determination appropriate? | Y | Y | Y | Y | Y | Y | Y | Y | PY | Y | PY | Y |
| **Domain 3 risk of bias** | **Unclear** | **Unclear** | **Unclear** | **Unclear** | **Unclear** | **Unclear** | **Unclear** | **Unclear** | **Unclear** | **Unclear** | **Unclear** | **Unclear** |
| **Applicability - Outcome** | **Low** | **Unclear** | **Low** | **Low** | **Low** | **Low** | **Unclear** | **Low** | **Low** | **Low** | **Low** | **Low** |
| **Domain 4 - Analysis** | | | | | | | | | | | | |
| 4.1 Were there a reasonable number of participants with the outcome? | PN | Y | PN | PY | PY | PY | PY | PY | Y | Y | PN | PN |
| 4.2 Were continuous predictors handled appropriately? | Y | Y | PY | Y | Y | Y | Y | Y | Y | Y | Y | Y |
| 4.3 Were all enrolled participants included in the analysis? | Y | NI | PY | Y | Y | Y | PY | PY | PY | NI | PY | Y |
| 4.4 Were participants with missing data handled appropriately? | Y | NI | PN | PY | PY | PY | PY | PY | PY | PY | PY | PY |
| 4.5 Was selection of predictors based on univariable analysis avoided? | Y | Y | NI | Y | Y | Y | Y | Y | Y | Y | Y | Y |
| 4.6 Were complexities in the data accounted for appropriately? | Y | PY | PY | PY | Y | PY | Y | Y | PY | PY | PN | PY |
| 4.7 Were relevant model performance measures evaluated appropriately? | Y | PY | PY | Y | Y | Y | Y | Y | Y | Y | Y | Y |
| 4.8 Were model overfitting and optimism in model performance accounted for? | PN | PY | PY | PY | PY | PN | PY | PY | PY | PN | PN | PY |
| 4.9 Do predictors and their assigned weights correspond to the reported multivariable analysis? | NI | NI | NI | NI | NI | NI | NI | NI | NI | NI | NI | NI |
| **Domain 4 risk of bias** | **High** | **Unclear** | **High** | **Low** | **Low** | **High** | **Low** | **Low** | **Low** | **High** | **High** | **High** |
| **Overall risk of bias** | **High** | **High** | **High** | **Unclear** | **Unclear** | **High** | **Unclear** | **Unclear** | **Unclear** | **High** | **High** | **High** |
| **Overall applicability** | **High** | **High** | **Unclear** | **High** | **High** | **High** | **High** | **High** | **Unclear** | **Unclear** | **High** | **High** |

Abbreviations: Y, Yes; PY, Probably Yes; PN, Probably No; N, No; NI, No Information. Risk-of-bias and applicability judgments are reported as Low, Unclear, or High.
